# Anthrax and Its Treatment with Sclavo's Serum

**Published:** 1907-09-28

**Authors:** 


### Points in Surgery.

### ANTHRAX AND ITS TREATMENT WITH SCLAVO'S SERUM.

Anthrax is a disease due to infection with a specific micro-organism, and incurred in the handling of wools, hides, and horsehair imported from certain foreign districts. For this reason it is known as "wool-sorters' disease" in the parts of England in which it is prevalent. It occurs in two main forms: local inoculation of the skin gives rise to a lesion known as "malignant pustule," while absorption of the bacilli or their toxins by the lungs or alimentary tract causes a general infection or "anthracæmia." Until quite recently the latter condition was regarded as inevitably fatal, and the mortality from malignant pustule, in spite of energetic surgical treatment by excision, was as high as 25 per cent.

### The Bacteriology of Anthrax.

The bacillus anthracis, the cause of this malady, is one of the largest of the known pathogenic micro-organisms, and is thus easily recognisable microscopically. If the blood of a diseased animal is examined, chains of bacilli of varying length can be found. The bacillus is sporogenous, when grown artificially in the presence of oxygen. No spores have so far been demonstrated in bacilli growing in the living tissues. It is to be supposed that spore formation is kept in reserve to continue the existence of the bacillus and preserve its species at those periods when it is existing in an unsuitable medium. The bacilli themselves are destroyed with moderate ease; boiling them for two or three minutes suffices to kill them, but the spores are peculiarly resistant; they have been known to remain alive even after a week's immersion in a 5 per cent. solution of carbolic acid. It is this power of resistance on the part of the spores which explains the fact that infection can be conveyed by hair from an infected animal many weeks, or even months, after its removal from the body.

### Malignant Pustule.

Malignant pustule may occur on any part of the body; but is naturally most common on the hands and forearms, since they are especially exposed to infection in the process of handling the hides. After these in order of frequency the face is most often attacked, presumably from being scratched by infected nails.

The pustule starts as a small red pimple at the site of inoculation. This spreads more or less rapidly, and becomes covered with one or more vesicles, whose serum contains active anthrax bacilli. The early stages are not associated with pain, a fact which induces patients suffering from this disease to take no notice of it, and to defer seeking medical advice until they are frightened into doing so by the progressive nature of the local lesion. By this time the prognosis is usually very grave, and the chance of checking the disease is sensibly diminished. After a varying period, usually from one to three days, the base of the pustule becomes much infiltrated, and is surrounded by a row of smaller vesicles. There is also very marked œdema of the tissues round the pustule, which is a characteristic feature of the disease, and may itself endanger the life of the patient: for instance, when the local lesion is situated in the neck. At the same time the central part of the pustule necroses and forms a greyish-black slough. These later stages are associated with much pain and pyrexia, and the general constitutional symptoms of acute inflammatory disturbance. The lymphatic glands which drain the site of inoculation are enlarged at an early date. In some 75 per cent. of cases the disease is limited to the local manifestations, which clear up gradually, but in the other 25 per cent. death ensues from the effects of acute toxæmia.

### Its Treatment.

Until quite recently the only form of treatment for malignant pustule consisted of measures which had for their object its removal or destruction by the knife or cautery; on the ground that the infection was a local one, and that if the bacilli could be removed entirely before their toxins were disseminated, the disease could be arrested. It is true that these methods reduced the death-rate somewhat from that of the days of purely conservative treatment by rest, fixation, and elevation of the affected part with the local application of a mercury ointment.

On the other hand, cases occurred in which the disease seemed to progress more rapidly after the removal of the focus and the surrounding tissues, presumably because some natural immunising body had been also removed in the process, or perhaps because bacilli were enabled to enter the blood by way of the cut vessels. In any case it is obvious that excision of the pustule is far from being a satisfactory treatment in all cases, since there are situations where, for anatomical reasons, it is impossible. Malignant pustule of the eyelid is comparatively common, and excision in such a case would lead at the best to frightful deformity.

### The Serum Treatment.

For the successful treatment of anthrax by a serum which shows prophylactic and curative powers against the disease, we are indebted to Professor Sclavo, of the University of Siena. After prolonged experiments, he has found that he can produce an active curative serum from asses which have been immunised against the disease. The serum is injected into the subcutaneous tissues of the abdominal wall as soon as the disease is recognised, and a big initial dose is given (40 cubic centimetres). In many cases this is sufficient to arrest its course, especially if the disease is taken early, and the patients recover without cessation of work for more than a day or two. By this means the death-rate from malignant pustule has been reduced in Italy from 25 to 5 per cent.

A fair number of cases have now been treated with this serum in England; in some of the cases excision was also performed, but in others this was impossible, for anatomical reasons. Mr. Stretton, of Kidderminster, reported such a case in the *Lancet* (May 27, 1905), and he remarked at the time: "The case was certainly a bad one, and from my experience I should have expected a fatal result. How far this was averted by the use of the serum it is impossible to say, but the results are so far very encouraging." As far as our knowledge goes at present it seems that no case of malignant pustule need be fatal if a large initial dose of serum is given at an early stage, while some very desperate cases have been saved. Its use reduces the destruction of tissue to a minimum; and it is the only treatment which gives any reasonable prospects of success in cases of generalised infection.

